# The Role of Glucagon-like Peptide-1 Receptor Agonists in Alzheimer’s and Parkinson’s Disease: A Literature Review of Clinical Trials

**DOI:** 10.3390/life15121893

**Published:** 2025-12-11

**Authors:** Joanna Pilśniak, Julia Węgrzynek-Gallina, Błażej Bednarczyk, Aleksandra Buczek, Aleksandra Pilśniak, Tomasz Chmiela, Agnieszka Jarosińska, Joanna Siuda, Michał Holecki

**Affiliations:** 1 Student Scientific Society, Department of Internal Medicine, Autoimmune Diseases and Diabetology, Faculty of Medical Sciences in Katowice, Medical University of Silesia, 14 Medykow St., 40-752 Katowice, Poland; 2 Department of Neurology, Faculty of Medical Sciences in Katowice, University Clinical Centre Prof K. Gibinski, Medical University of Silesia, 14 Medykow St., 40-752 Katowice, Poland; 3 Student Scientific Society, Department of Neurology, Faculty of Medical Sciences in Katowice, Medical University of Silesia, 14 Medykow St., 40-752 Katowice, Poland; 4 Department of Internal Medicine, Autoimmune Diseases and Diabetology, Faculty of Medical Sciences in Katowice, Medical University of Silesia, 14 Medykow St., 40-752 Katowice, Poland

**Keywords:** GLP-1RA, antidiabetic drugs, neurodegenerative diseases, Alzheimer’s disease, Parkinson’s disease

## Abstract

Glucagon-like peptide-1 receptor agonists (GLP-1RAs) are widely used in the treatment of type 2 diabetes and obesity due to their metabolic effects. Emerging evidence suggests they may also have neuroprotective effects, indicating their potential as disease-modifying therapies in neurodegenerative disorders such as Alzheimer’s disease (AD) and Parkinson’s disease (PD). Preclinical studies in animal models have demonstrated that GLP-1RAs can reduce neuroinflammation, oxidative stress, neuronal apoptosis, and pathological protein aggregation, while enhancing glucose metabolism and mitochondrial function. This narrative review analyzed results from human clinical trials evaluating GLP-1RAs in AD and PD, based on a search of four databases (Web of Science, Medline, Embase, and Clinical Trials). The analysis included eleven studies. In AD, clinical trials suggest that GLP-1RAs such as liraglutide and semaglutide may enhance brain glucose metabolism, facilitate glucose transport across the blood–brain barrier, and benefit neuronal networks. However, most studies did not demonstrate improvements in cognitive functions or radiological markers. Short-term clinical trials of GLP-1RAs, including exenatide and lixisenatide, demonstrated promising effects on motor and selected non-motor symptoms in patients with PD, but their disease-modifying effects remain unproven. GLP-1RAs showed a favorable safety profile. Despite promising findings, small study populations, heterogeneous protocols, and short observation periods limit definitive conclusions. Further larger, long-term studies are needed, particularly to clarify the risk–benefit balance, weight control, and long-term outcomes.

## 1. Introduction

Glucagon-like peptide-1 (GLP-1) is a 30-amino acid peptide hormone secreted by intestinal L-cells after food intake [1,2]. This incretin hormone plays a crucial role in maintaining metabolic homeostasis. The main functions of GLP-1 are to enhance insulin secretion from pancreatic β-cells, delay gastric emptying, and suppress glucagon secretion, thereby limiting postprandial glucose excursions, reducing appetite, and resulting in long-term weight loss [3,4]. Due to the abovementioned mechanisms, GLP-1 receptor analogs (GLP-1RA) are often used to treat type 2 diabetes mellitus (T2DM) and obesity.

Endogenous GLP-1 has a short half-life (1–2 min), as it is rapidly degraded by dipeptidyl peptidase-4 (DPP-4) and undergoes renal elimination. DPP-4 inhibitors (DPP-4i) are a class of oral hypoglycemic medications that work by increasing incretin levels. It is important to note the potential use of DPP-4i in the treatment of neurodegenerative diseases. Although previous reports indicated that DPP-4i cannot cross the blood–brain barrier (BBB), suggesting that the neuroprotective effect of DPP-4i may only be conferred indirectly through an increase in peripheral blood GLP-1 [5,6]. However, recent studies have emerged that offer a novel perspective, demonstrating the potential for omarigliptin to cross the BBB [7]. Due to the short plasma half-life of native GLP-1, several GLP-1RAs have been developed for the treatment of T2DM [2], for example, short-acting analogs such as lixisenatide (half-life ~3 h), intermediate-acting analogs such as liraglutide (half-life 13–15 h), and long-acting analogs such as semaglutide (half-life 1 week) [3].

GLP-1 is produced peripherally in the ileum, from where it is released into the bloodstream. It can cross the BBB or communicate through the gut–brain signaling pathway using the vagus nerve. It is also produced centrally in limited areas of the brain, including the nucleus of the solitary tract and the olfactory bulb [8]. It exerts its effects by binding itself to its transmembrane receptor, which is predominantly expressed in the beta cells of the pancreatic islets and throughout the brain [9]. In a brain mapping study by Tulika Gupta et al., GLP-1 expression was found in most cortical areas, with the highest levels in the frontal, prefrontal, and parietal cortices, as well as in the diencephalon and brainstem. Interestingly, a decrease in GLP-1 expression was observed in most of these areas after the fifth decade of life [10]. Preclinical studies in animal models have demonstrated the neuroprotective and anti-inflammatory properties of GLP-1RAs and confirmed their therapeutic potential for neurodegenerative diseases such as Alzheimer’s disease (AD) and Parkinson’s disease (PD) [11,12,13]. Currently, AD and PD are the two most common neurodegenerative diseases, affecting approximately 50 million and 10 million people worldwide, respectively, and these numbers are expected to rise to 150 million and 12 million by 2050 [14].

AD is caused by the deposition of β-amyloid plaques and neurofibrillary tangles of hyperphosphorylated tau, which leads to the atrophy of neurons and their connections, resulting in the deterioration of the cognitive function, behavioral changes, and loss of independence in the advanced stages of the disease [15]. PD is characterized by motor dysfunction, including bradykinesia, rigidity, resting tremor, and postural reflex disturbances. Non-motor symptoms of PD include dementia, sleep disorders, olfactory disturbances, dysfunction of the autonomic system, including gastrointestinal dysfunction, urinary incontinence, orthostatic hypotension, thermoregulatory disturbances, as well as anxiety and depressive episodes [16]. The pathological mechanism of PD involves the formation of intra-neuronal Lewy bodies, deposits of the α-synuclein protein, which lead to the loss of cells in the substantia nigra, the area of the brain responsible for synthesizing the neurotransmitter dopamine [17]. Numerous studies have shown the interplay between PD and diabetes [18,19,20,21,22]. Several mechanisms, including disturbances in insulin signaling, insulin resistance, oxidative stress, neuroinflammation, and aggregation of misfolded proteins observed in both T2DM and PD, have been proposed to link these two age-related diseases [18,19]. In addition, autonomic motor symptoms in PD might contribute to a higher prevalence of prediabetes and worse glycemic profile [20,21,22].

These observations have led to the conclusion that those drugs used to treat T2DM which target a common pathophysiological mechanism could present a potential new treatment option in neurodegenerative diseases [23,24]. Considering this and the positive results from studies in animal models [25,26], further research was warranted to explore this topic in clinical trials involving humans [27]. This review aims to gather information from human clinical trials to assess the potential of GLP-1RAs as a disease-modifying agent, influencing the neurodegenerative process rather than only alleviating symptoms, for the treatment of AD and PD. This review extends the literature by focusing on human clinical trials, addressing a gap left by earlier reviews that rely mainly on preclinical evidence. It also offers a comparative analysis of Alzheimer’s and Parkinson’s disease, synthesizing trial outcomes across both conditions to highlight shared and divergent therapeutic implications. By examining methodological differences across studies, this review provides a clearer framework for interpreting current evidence and identifying priorities for future research.

## 2. The Mechanism of Actions of GLP-1RAs

### 2.1. GLP-1RAs in Alzheimer’s Disease

An example of a neurodegenerative disease where GLP-1RAs can have a potential therapeutic use is AD. Zhang et al. tested the influence of semaglutide on cognitive functions of AD mice, administrating this GLP-1RA for 6 months. After 3 months, they were assessed by the Barnes maze test. After the 5th day of assessment, significant improvement in learning abilities and nest building scores was observed compared the control group [28]. Study conducted by Carranza-Naval et al., also proved that liraglutide treatment can improve cognitive functions in the AD mouse models. Moreover, there was a reduction in amyloid plaque volume in the cortex, hyperphosphorylated Tau protein and finally significant reduction in microglia activation [25].

**Figure 1:**
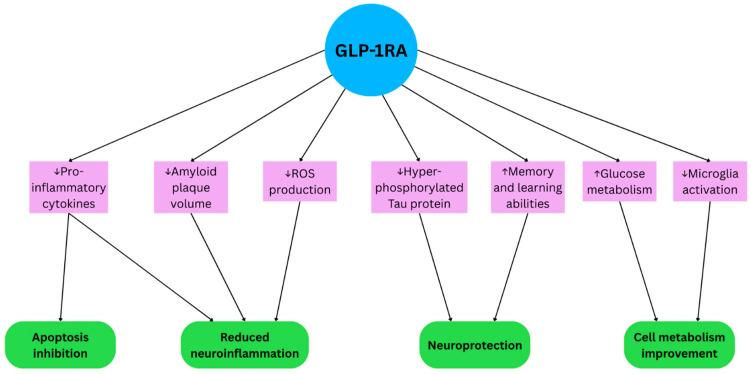
The mechanism of action of GLP-1 analogs in Alzheimer’s Disease.

The aggregation of amyloid beta can also damage mitochondria, leading to increased production of reactive oxygen species (ROS) (Figure 1) [29]. However, the study performed by Xie et al. showed a decrease in the overproduction of ROS in the AD rats treated with liraglutide [26]. Similar results were found by Gwangho Yoon et al., who investigated the anti-inflammatory effects of GLP-1RAs in the microglial and neuronal cells of rats exposed to lipopolysaccharide (LPS). In the microglial treated with GLP-1, the secretion of pro-inflammatory cytokines and chemokines was reduced compared to neurons treated with LPS alone. Furthermore, GLP-1 improved neurite complexity, dendritic spine morphogenesis, and spine development in TNF-α-treated primary cortical neurons [30].

Tirzepatide, a GLP-1 and GIP receptor agonist, improved glucose metabolism in the hypothalamus of APP/PS1 mice. Yang et al. administered tirzepatide intraperitoneally once a week for 8 weeks, which increased the expression of hexokinase, glucose transporters, and other key factors in the glucose metabolism pathway, compared to untreated APP/PS1 mice (Figure 1) [31].

In conclusion, preclinical studies indicate that GLP-1RAs may have the potential to modify disease progression. However, further investigation in human studies is required before they can be considered as a potential alternative to current symptomatic treatments for AD.

### 2.2. GLP-1RAs in Parkinson’s Disease

The study conducted by Dürdane Aksoy et al. showed that a group of rats with rotenone-induced PD had a higher total number of neurons after 28 days of exenatide treatment than the control group. Tyrosine hydroxylase levels, a marker used to detect dopamine, were also significantly higher in the exenatide-treated rats [32]. In another study Exendin-IV was injected intraperitoneally twice a day for 4 or 8 weeks in a PD rat model of α-synucleinopathy. By the end of the treatment period, it attenuated dopaminergic neurons loss and almost fully prevented further neuronal loss [33]. The mechanism underlying these effects has been investigated in several studies [23,34,35,36].

**Figure 2:**
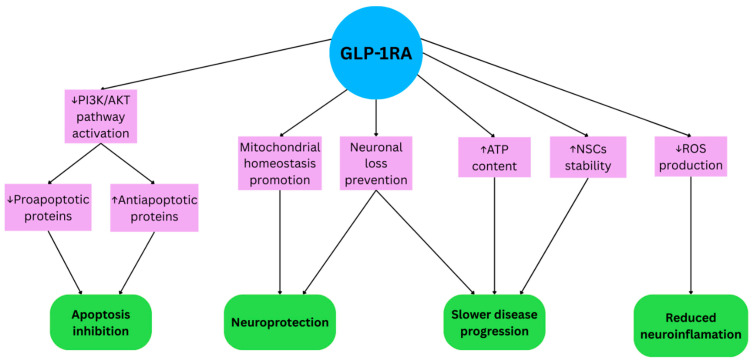
The mechanism of action of GLP-1 analogs in Parkinson’s Disease.

GLP-1RAs may reduce neuroinflammatory damage in brain cells and prolong cell survival by inhibition of NF-κB [23]. They contribute to the activation of the phosphoinositide 3-kinase (PI3K)/protein kinase B (Akt) pathway, which plays a relevant role in the process of cell survival (Figure 2). A study conducted by Zhu et al. showed that liraglutide activated the PI3K/AKT pathway in the neuronal cells of mice damaged by ischemia, which increased the concentration of anti-apoptotic proteins such as Bcl-2 and Bcl-xl (Figure 2). In addition, the expression of pro-apoptotic proteins belonging to the Bax and Bad families was reduced (Figure 2) [34].

GLP-1RAs play important role in mitochondrial homeostasis promotion (Figure 2). The study by Wang et al. showed that a reduction in ROS production was observed in the exenatide-treated group of PD mouse model compared with controls (Figure 2) [36]. Tian et al. proved that treatment of MPTP-induced PD mice with tirzepatide or semaglutide for 4 weeks can reduce of Drp-1 protein concentration (key marker of mitochondrial fission) in this group. Treatment with tirzepatide also helped to restore ATP content in the MPTP-impaired brain tissue, which can prevent neuronal loss the hallmark of the PD (Figure 2) [37].

In the study conducted by Song et al. 6-OHDA mice group received transplanted neural stem cells (NSCs) and semaglutide (Figure 2). The rodents showed improved motor function. They also showed improvement in survival and differentiation of stem cells compared to the vehicle trail and group treated with NSCs alone too. Transplanted cells migrated to the surrounding brain areas, which was not present in the group given NSCs only [38].

Preliminary evidence from animal and cell studies suggests that GLP-1RAs protect neuronal cells from damage by modulating inflammatory mechanisms and pathways leading to apoptosis. This helps preserve neuronal integrity but also reduces neuronal loss, particularly of dopaminergic neurons, providing a potential basis for use in PD therapy.

## 3. Materials and Methods

This article presents published research providing information on the use of GLP-1RAs in treating neurodegenerative diseases. This paper presents a narrative review. The literature search was conducted on 26 October 2024, following strictly the established inclusion and exclusion criteria. Four databases (Web of Science, Medline, Embase, Clinical Trial) were searched by the authors using the following keywords: ((GLP-1) OR (glucagon-like peptide-1)) AND ((neurodegeneration) OR (Alzheimer) OR (frontotemporal dementia) OR (Parkinson) OR (atypical parkinsonism) OR (multiple system atrophy) OR (dementia with Lewy bodies) OR (corticobasal dementia) OR (progressive supranuclear palsy)).

The results were not limited to any time frame. Experimental studies in English and Polish describing the use of GLP-1RAs in the treatment of neurodegenerative diseases such as PD, atypical Parkinsonisms including Multiple System Atrophy (MSA), Dementia with Lewy Bodies, Corticobasal Dementia, Progressive Supranuclear Palsy, AD, and Frontotemporal Dementia were included.

To avoid bias in manuscripts, the following inclusion and exclusion criteria were used in this search (see Table 1).

All qualified studies were also screened for eligibility by independent authors.

The initial search identified 2010 articles. Of these, 1122 of these were automatically identified as duplicates by the software tool and excluded. 888 were reviewed by the authors, and 58 were selected for full reading. Twelve eligible studies were included in the analysis. Five completed studies with published results focused on AD, while seven focused on PD. Five ongoing GLP-1RA studies focused on AD, while seven focused on PD.

### 3.1. Results of Clinical Trials with GLP-1RAs in AD

A large number of preclinical studies suggest potential therapeutic use of GLP-1RA in the treatment of AD [39,40]. This review aims to determine whether these findings are reflected in human clinical trials.

In 2016, Michael Gejl et al. conducted a clinical trial involving 38 patients with long-term AD, in which the authors assessed cerebral glucose metabolism (CMRglc) using [18F] FDG and fibrillar Aβ deposition with [11C] PIB PET. The authors also assessed changes in the cognitive function using the Wechsler Memory Scale (WMS-IV) [27]. Patients were randomized into two groups: one group received the GLP-1RA liraglutide (*n* = 18), while the other group received a saline placebo (*n* = 20) for 26 weeks. After six months, placebo-treated participants showed a significant decrease in CMRglc in the precuneus, parietal, temporal, and occipital cortices, as well as in the cerebellum. Treatment with the GLP-1RA liraglutide prevented the decline in CMRglc. In addition, there was a significant reduction in body weight, systolic and diastolic blood pressure, and fasting plasma glucose levels in the GLP-1RA group compared with the placebo group. However, no significant differences were found between the groups in terms of amyloid deposition or cognitive function as measured by the WMS-IV. The most commonly reported side effects were of gastrointestinal nature, particularly nausea. It should be noted that the small sample size (38 patients) and the short duration of the study (26 weeks) limited the ability to draw definitive clinical conclusions [27]. Nevertheless, liraglutide prevented the decline in CMRglc, which could potentially slow disease progression. This suggests that GLP-1RAs may have metabolic–neuroprotective effects, and this study encourages further research in this area.

One year later, Michael Gejl et al. investigated the mechanism by which liraglutide prevents the decline in CMRglc [41]. A GLP-1RA was expected to increase blood-brain glucose transfer in a time-dependent manner. In both groups, estimates of blood-brain glucose transfer (T max) correlated inversely with the duration of AD. Moreover, CMRglc estimates correlated positively with cognition. This finding confirmed the expected natural progression of the disease in the study population. The study showed that treatment with a GLP-1RA significantly increased blood-brain glucose transfer (T max) in the cerebral cortex, from 0.72 to 1.1 μmol/g/min. This is comparable to the estimated T max in healthy volunteers of 1.022 μmol/g/min. These results support the hypothesis that treatment with a GLP-1RA restores glucose transport at the blood–brain barrier, brain glucose availability, and neuronal metabolism [41].

In 2018, a double-blind, placebo-controlled study was conducted to investigate the effects of liraglutide administration on neurons in cognitively normal, late middle-aged individuals with subjective cognitive complaints [42]. The study lasted for 12 weeks. It was found that higher fasting plasma glucose (FPG) levels at baseline were associated with reduced connectivity between bilateral hippocampal and medial frontal structures. An inverse correlation was found between insulin resistance (measured by FPG) and connectivity between the bilateral hippocampus and a cluster centered in the left anterior cingulate. Furthermore, in the liraglutide-treated group, there was an increase in internal connectivity between the bilateral hippocampus and three clusters centered in the left middle frontal gyrus, bilateral posterior cingulate, and left lateral occipital cortex, respectively. Although successfully reversing the loss of connectivity appears to help control disease progression, no improvement in cognitive function was observed with liraglutide in this study [42].

Roger J Mullins et al. conducted an 18-month clinical trial with exenatide on 27 patients with early AD. The researchers sought to determine response to treatment based on clinical, cognitive, magnetic resonance imaging (MRI), and biochemical biomarker scores [43]. Neuropsychological outcomes (including Mini Mental State Examination, ADAS-cog, and CDR-SOB) demonstrated minimal differences between the exenatide and placebo groups after 18 months. Furthermore, an improvement in the digit-span forward task was observed in the exenatide group after six months, reflecting attention and short-term memory. This task is sensitive to mild cognitive impairment but non-specific for dementia. After 18 months, MRI demonstrated the progression of GM atrophy and thinning of the cerebral cortex (mainly in the medial and lateral temporal and parietal areas), but without significant differences between the two groups. No significant treatment effects were observed on biomarkers in CSF (Aβ42, total tau, p181-tau), plasma (Aβ42, Aβ40), and plasma neuronal extracellular vesicles (p-181tau, IRS-1, Aβ40). Except for Aβ42 in extracellular vesicles, which decreased significantly over time in the exenatide-treated group. These findings indicate a decrease in the severity of brain amyloidosis; exenatide was a safe and well-tolerated treatment option for early AD. The treatment group experienced mainly gastrointestinal side effects without any serious problems. The study was not conclusive due to early termination by the sponsor, so no firm conclusions can be drawn [43].

The next study, by A. Dei Cas et al., used the long-acting GLP-1RA exenatide (2 mg once-weekly as a subcutaneous injection) in patients with mild cognitive impairment (MCI) and found no beneficial effect on cognitive performance. In this trial involving 32 patients, no significant time-dependent (*p* = 0.65) or treatment-dependent (*p* = 0.17) differences in cognitive function assessment by ADAS-Cog scores were observed. Of note, the exenatide-treated group showed improved metabolic parameters compared to untreated individuals. The reduction in fasting blood glucose and body weight was statistically significant [44].

A review of the results of all clinical trials previously cited in this study reveals a mixed outcome, which should be interpreted with caution. Some studies suggest that GLP-1RAs therapy may have the potential to modify disease progression. Improvements in metabolic or neuronal processes, including preserved CMRglc [27], increased blood-brain glucose transfer [41], and enhanced hippocampal connectivity, are observed more frequently than actual clinical improvements in cognitive function.

It should be noted that the number of studies is limited and they are heterogeneous. The studies included patients diagnosed with AD, patients with MCI or normal cognition with subjective complaints. Different GLP-1RAs were administrated in non-standardized doses. Moreover, the duration of individual studies and endpoints varied. In turn, due to the limitations of the studies included, further studies with larger samples are needed to draw more concrete conclusions. The summary of all presented studies was prepared in Table 2.

Similar studies are still ongoing, as listed in Table 3. Some of them are multicenter studies with larger study groups, which may open up new perspectives on the topic.

### 3.2. Results of Clinical Trials with GLP-1RAs in Parkinsonism

Although the number of completed clinical studies is still limited, the initial results seem promising. Encouraged by the neuroprotective and neurorestorative effects of GLP-1RAs observed in animal models [25,45], in 2013, Aviles-Olmos et al. conducted a clinical study with exenatide regarding its tolerability and safety. This research provided preliminary indications for further studies [46]. The authors recruited patients with moderate PD to the experimental group which received twice-daily subcutaneous injections for 12 months. The first outcome assessed by the commonly used motor symptoms scale—Movement Disorders Society-Unified Parkinson’s Disease Rating Scale (MDS-UPDRS) part 3—revealed improvement in the exenatide group of 4.9 points compared to controls (95% CI, 0.3–9.4; *p* = 0.037). After 14 months, the difference was slightly lower, at 4.4 points (95% CI, 0.2–8.7; *p* = 0.042). Moreover, the authors observed a reduction in rigidity and the cognitive function. The tolerability and safety of the therapy used were satisfactory. The adverse events reported by the patients were mainly gastrointestinal in nature. Weight loss and nausea were more common in the exenatide group. Patients receiving exenatide lost 3.2 ± 3.9 kg over 12 months. No clinically significant changes in ECG, hematologic, or biochemical parameters were observed [46].

Further studies were performed by Athauda et al. in 2017 [24,47,48,49]. Patients with moderate PD were treated with exenatide for 48 weeks, resulting in the significant improvement of motor symptoms measured by MDS-UDPRS part III, greater in the experimental group by −4.3 points (−7.1 to −1.6; *p* = 0.0026). After 60 weeks, when the treatment was discontinued, the difference became smaller. In this study, there were no significant differences in cognitive functions between the exenatide and placebo groups [24]. However, an extended post hoc analysis of non-motor symptoms revealed a significant improvement in the exenatide group compared to controls in mood scores assessed with various questionnaire methods [48].

The same authors performed a further analysis of factors predicting a good response to exenatide treatment and showed that patients whose disease duration was less than four years showed a trend towards a lower disease burden. A poorer response was observed when the disease duration was over ten years [47]. High responders had no postural instability or speech impairment at baseline. There were no significant differences between the treatment group and disease duration, insulin resistance, motor phenotype, presence of MCI, or obesity according to motor symptoms [47].

In a study by Ahauda et al. [49], the researchers investigated whether the participants in the exenatide-PD trial showed increased activity in the insulin and Akt signaling pathways in the brain, which are known to play a key role in the pathogenesis of PD. It is assumed that impaired insulin and Akt signaling in the brain contributes to the development and progression of Parkinson’s disease. The study, therefore, aimed to investigate whether exenatide can influence these mechanisms. After 48 weeks of exenatide therapy, the authors found higher insulin receptor substrate 1 (IRS-1) levels in the extracellular vesicles of neurons and increased expression of phosphorylated mTOR and PI3K/AKT. These results suggest that exenatide affects insulin, Akt, and mTOR signaling pathways in the brain, which helps explain the clinical results of the exenatide PD study. These findings provide support for the conclusion that exenatide may have a disease-modifying effect. They also suggested that exosome-based biomarkers could be used as objective measures of how well drugs targeting brain pathways are working in clinical trials.

In the study by McGarry et al. [50] on early-stage PD treated with NLY01, a brain-penetrant, pegylated, longer-lasting version of exenatide was administered to patients in 36 weeks in two different doses; the study did not show differences in motor and non-motor symptoms between treated and control branches. The treatment was generally well tolerated; the most common adverse effect was nausea (58% at higher doses and 39% at lower doses).

Another early PD study of lixisenatide by Meissner et al. [51] showed a significant improvement in MDS-UPDRS part III in the experimental group after 12 months of treatment. As in the exenatide studies, the most common adverse events were gastrointestinal in nature. Nausea was reported by 46% of patients, vomiting by 13%, and gastroesophageal reflux by 8%. Only a few patients reported weight loss. The incidence of serious adverse events was similar in the intervention and control groups. The detailed data describing the studies are shown in Table 4.

Further clinical trials are ongoing to investigate the effects of GLP-1RA therapy (Table 5). Most of them have evaluated exenatide or its derivatives. The exenatide clinical trial NCT04232969 is in the 3rd phase of research. A single study (NCT03659682) is focused on semaglutide.

Apart from the mentioned PD studies, there is ongoing research regarding the role of exenatide in MSA, an atypical Parkinsonism that is clinically characterized by a combination of Parkinsonian symptoms, severe autonomic dysfunction, and cerebellar ataxia (Table 5) [52]. MSA is a rapidly progressive disease in symptoms that leads to early disability. There are no effective symptom- or disease-modifying treatments for this disorder [53]. No other studies on atypical parkinsonism were found.

Available preliminary results from clinical studies have concerned improvement mainly in motor symptoms in PD patients, especially in younger groups without serious adverse events. To assess the efficacy of this experimental therapy, a larger and longer observation is needed, taking into account confounding factors. Assuming that these results are particularly relevant, it is necessary to determine the direction of further research that may allow the expansion of the pool of antiparkinsonian drugs one day.

**Table 4:** Description of the study group and results in GLP-1RAs clinical trials in PD patients.

Number	Citation	Study Group, (*n*)	Age, Years	Intervention (Drug and Dose)	Clinical Trial Duration	Results
1	Aviles-Olmos et al., 2013 [46]	Exenatide (*n* = 20) Conventional PD medication (*n* = 24)	Exenatide 51.6 (7.8) Conventional PD medication 48.4 (7.4)	Exenatide, 5 μg twice a day for 1 month and 10 μg twice a day for 11 months. Total duration of treatment: 12 months	14 months	Improvement of the exenatide group compared to controls in MDS-UPDRS part 3 OFF state after 12 months and 14 months. Improvement of cognitive function in the Mattis dementia rating scale–2 at 14 months compared with deterioration in control patients.
2	Athauda et al., 2017 [24]	Exenatide (*n* = 31) Placebo (*n* = 29)	Exenatide 61.6 (8.2) Placebo 57.8 (8.0)	Exenatide 2 mg	48 weeks	Improvement of the exenatide group compared to controls in MDS-UPDRS part 3 OFF state after 48 and 60 weeks. No cognitive improvement.
3	Athauda et al., 2019 [47]					Post hoc analysis of factors predicting response assessed that more disease severity and longer disease duration may benefit less from exenatide than patients with less severity and shorter duration.
4	Athauda et al., 2018 [48]					Improvement in emotional dysfunction/depression across the NMSS, MDS-UPDRS Part 1 and MADRS assessment. Improvement in the “well-being” domain of PDQ-39. No statistical significance in cognitive functions and other non-motor symptoms.
5	Athauda et al., 2019 [49]					Higher levels of insulin receptor substrate 1 (IRS-1) phosphorylation at tyrosine sites and higher levels of phosphorylated mTOR and phosphoinositide-3-kinase/Akt (PI3K/AKT) expression in the intervention group compared to the controls.
6	Meissner et al., 2024 [51]	Lixisenatide (*n* = 78) Placebo (*n* = 78)	Lixisenatide 59.5 ± 8.1 Placebo 59.9 ± 8.4	Lixisenatide 20 μg	48 weeks	Lixisenatide modestly reduced motor disability progression in patients with early PD as compared with a placebo but had gastrointestinal side effects.
7	McGarry et al., 2024 [50]	NLY01 2.5 mg (*n* = 85) NLY01 5 mg (*n* = 85) Placebo (*n* = 84)	NLY01 2.5 mg 62.1 ± 9.0 NLY01 5 mg 60.6 ± 10.0 Placebo 61.8 ± 8.1	Exenatide and polyethylene glycol (NLY01) (2.5 mg/5 mg/placebo)	36 weeks	NLY01 did not differ between the active group compared with placebo.

**Table 5:** Ongoing GLP-1RAs clinical trials in Parkinsonism.

Number	Condition	Title	ClinicalTrials.gov ID	Phase	Status	Intervention (Drug and Dose)	Clinical Trial Duration
1	Parkinson’s disease	Effects of Exenatide on Motor Function and the Brain	NCT03456687	Phase 1	Completed	Exenatide 2 mg once a week	12 months
2	Parkinson’s disease	Exenatide Treatment in Parkinson’s Disease	NCT04305002	Phase 2	Unknown status	Exenatide 2 mg once a week	18 months
3	Parkinson’s disease	GLP1R in Parkinson’s Disease	NCT03659682	Phase 2	Not yet recruiting	Semaglutide 1 mg once a week	48 months
4	Parkinson’s disease	Exenatide Once Weekly Over 2 Years as a Potential Disease Modifying Treatment for Parkinson’s Disease	NCT04232969	Phase 3	Active, not recruiting	Exenatide extended release 2 mg once a week	96 weeks
5	Parkinson’s disease	GLP1R in Parkinson’s Disease	NCT03659682	Phase 2	Not yet recruiting	Semaglutide 1 mg once a week	48 months
6	Early Parkinson’s disease	SR-Exenatide (PT320) to Evaluate Efficacy and Safety in Patients With Early Parkinson’s Disease	NCT04269642	Phase 2	Unknown status	SR-exenatide (PT320) 2 mg once a week PT320 2.5 mg every 2 weeks	48 weeks
7	Multiple system atrophy	Exenatide Once-weekly as a Treatment for Multiple System Atrophy	NCT04431713	Phase 2	Unknown status	Exenatide	12 months

## 4. Clinical Implications of the Results

Some clinical trials investigating the potential benefits of GLP-1RAs in AD have shown preliminary positive results that may indicate slowing the progression of the disease. Michael Gejl et al. found that treatment with the GLP-1RA liraglutide prevented the decline in CMRglc compared to placebo [27]. Another study by Michael Gejl et al. showed that treatment with liraglutide significantly increased glucose transfer from the blood to the cerebral cortex, with results comparable to those in healthy volunteers [41]. In 2018, a study by Kathleen T. Watson et al. discovered that liraglutide improved the intrinsic connectivity of the default mode network [42]. However, no improvement in cognitive function was observed in any of the studies. A further study conducted on patients diagnosed with mild cognitive impairment did not show any significant improvement in cognitive performance [44]. Nonetheless, improved metabolic parameters were observed in the exenatide-treated group compared to untreated patients. The reduction in fasting plasma glucose levels and body weight was statistically significant. In a study by Michael Gejl et al. from 2016, there was a significant reduction in body weight, systolic and diastolic blood pressure, and fasting blood glucose in the GLP-1RA group compared to the placebo group [27].

GLP-1RAs have a generally good safety profile, with mainly mild to moderate gastrointestinal side effects, including nausea, loss of appetite, and weight loss. In Mullins et al. study of older participants (71.7 ± 6.9 years), 38% of those treated with exenatide reported nausea compared to none in the placebo group, gastrointestinal symptoms and loss of appetite were also significantly more common. Two participants who could not tolerate the higher dose returned to the lower dose, which was maintained throughout the study [43]. However, the incidence of nausea was similar to that observed in clinical trials in people with DM. In the Dei Cas et al. study of patients with MCI, gastrointestinal complaints were mild, though although 6 participants discontinued treatment with exenatide for this reason [44]. Overall, the safety profiles were consistent across these studies.

Individual studies have shown considerable heterogeneity. Three of the studies described used liraglutide in the same, gradually increasing doses up to a final dose of 1.8 mg, but their duration varied [27,41,42]. The studies by Geil et al. from 2016 and 2017 lasted 26 weeks [27,41], while the study by Watson et al. lasted only 12 weeks [42]. Mullis et al. tested the use of exenatide at a dose of 5 µg twice daily, with an increase to 10 µg twice daily, in an 18-month study. Dei Cas et al., on the other hand, used long-acting exenatide at a dose of 2 mg once a week in their study. In addition, the study populations varied considerably, including individuals at different stages of AD (long-term AD [27,41] or early AD [43]), as well as patients with MCI [44] and individuals with normal cognitive function reporting subjective complaints [42]. Due to the heterogeneity of the study populations, pharmacological agents used, dosing regimens, duration of intervention, the current state of knowledge does not allow for definitive conclusions to be drawn.

Diabetes is associated with an increased risk of dementia and is often present as a comorbidity in converters from MCI to dementia. It is important to identify those who are at an increased risk of accelerated cognitive decline early and to develop effective treatments [54]. Treatment with semaglutide, a GLP-1RA, has been shown to be associated with a reduced risk of first-time diagnosis of Alzheimer’s disease in patients with type 2 diabetes compared with other antidiabetic agents, including other GLP-1RAs. In addition, semaglutide was associated with significantly fewer prescriptions for AD-related medications [55].

The improved metabolic outcomes and safety of the therapy support the use of GLP-1RAs in the treatment of patients with T2DM, particularly those at risk of early development of cognitive dysfunction. In summary, while clinical trials have not demonstrated direct effects in AD or MCI, some beneficial effects are still observed in patients with T2DM.

Initial results from clinical trials evaluating the use of such GLP-1RAs as exenatide and lixisenatide in the treatment of Parkinson’s disease are promising. They show improvements in motor symptoms [24,46,51]. The most remarkable improvements were observed in patients with a shorter duration of disease, without balance disorders and speech disorders at the beginning of treatment [47]. Improvement was observed in some non-motor aspects as well [48]. This is a broad symptomatic effect of treatment. Moreover, as molecular research has revealed, GLP-1RAs has a positive effect on brain signaling pathways, especially insulin and Akt signaling pathways [49]. These findings suggest that this therapy may have a disease-modifying mechanism. However, interpretation of these findings should consider several potential confounding factors, including the stage of the disease evaluated in the study and gastrointestinal factors, which may influence tolerability and therapeutic response.

Clinical trials have included patients at different stages of the disease, which enables the assessment of the optimal timing for initiating therapy. Though some authors have demonstrated the efficiency of GLP-1RA in moderate stages of PD [24,47], the better response was observed in earlier stages with less advanced symptoms [47,51]. These findings highlight the potential benefits of optimizing treatment strategies at the initial stages of the disease. However, the disease stage itself constitutes an important confounder. Patients in the early stages typically progress more slowly and may have fewer comorbidities that affect treatment outcomes. Verifying a true disease-modifying effect therefore requires early treatment to be monitored using objective biomarkers. By comparison, symptomatic effects are easier to confirm in patients with already pronounced symptoms.

Although there have been no serious side effects of the therapy, gastrointestinal side effects can be a serious problem in Parkinson’s disease, especially in relation to weight loss. It is known that weight loss is the main concern of this group of patients [56,57]. Some sources indicate that this can occur several years before diagnosis [56]. It can occur with non-motor gastrointestinal symptoms such as constipation, dysphagia, delayed gastric emptying, or as a result of taking levodopa [57], and could lead to higher comorbidity, poor physical and mental function, frailty, and increased mortality [56]. There is evidence that some PD therapies, such as deep brain stimulation (DBS), can lead to weight gain [58]. The beneficial aspect of this effect should be considered in the context of cardiovascular risk, but data are currently limited [59].

Given the importance of weight management in PD, especially considering the negative impacts of weight loss, it is crucial to evaluate whether the use of GLP-1RAs provides more benefits than drawbacks. While the initial results of GLP-1RA therapies for PD are promising, particularly in improving motor symptoms and non-motor aspects, careful consideration of gastrointestinal side effects, particularly related to weight loss, is essential to determine whether the benefits outweigh the potential risks. Furthermore, gastrointestinal effects and weight loss may influence tolerability, adherence, and ultimately treatment response. These variables were not consistently controlled across studies, representing a significant confounding factor.

Interpretation of the available trials requires caution, not only regarding side effects but also with respect to methodology. A major limitation was the small sample size. Studies on AD patients included groups of no more than 30 participants [27,41,42,43,44], which may have contributed to the lack of observed clinical effects. In one AD trial, the relevant limitation was the early termination of the study by the sponsor, which made it impossible to draw reliable conclusions [43]. Similar limitations resulting from the small size of the groups were noted in PD trials [24,46,47,48,49], although some studies were larger [50,51]; the sample sizes still remain relatively small. Future studies should aim to include larger cohorts to provide more reliable evidence.

Another important consideration is the heterogeneity of the studies. Across clinical trials on PD, different substances were used. Aviles-Olmos et al. and Athauda et al. evaluated exenatide [24,46,47,48,49], Meissner et al. [51] investigated lixisenatide, and McGarry et al. examined NLY01 [50]. This diversity of compounds limits the ability to draw consistent and comparable conclusions. Variations in the dosage of GLP-1RAs were also observed. Although both Aviles-Olmos et al. and Athauda et al. examined exenatide, the former administered 5–10 μg twice daily, whereas the latter used 2 mg once weekly [24,46]. Treatment durations were heterogeneous as well [24,46,47,48,49], resulting in different time points for assessing treatment outcomes. Furthermore, the studies assessed a wide range of clinical and biochemical endpoints—including motor outcomes [24,46], cognitive performance [28,48], mood and non-motor symptoms [48], and biomarker changes [59]—which further increases outcome heterogeneity and complicates cross-study comparisons. Future research should focus on systematically comparing these factors and standardizing study protocols.

A significant goal for neurodegenerative disease therapies is finding a disease-modifying treatment that influences the course of the disease. Based on the available clinical evidence on AD therapy [27] with GLP-1RAs, the conclusions are limited, and many questions remain. This treatment may present some metabolic and neuroprotective effects [27,41], though in one study, a possible disease-modifying effect was observed [42]. PD trials demonstrated that GLP-1RAs may have disease-modifying potential through their effects on insulin, Akt, and mTOR signaling pathways in the brain, which are involved in the neurodegenerative process [49]. However, this has not been confirmed and still requires further evidence and clarification.

## 5. Conclusions

Neurodegenerative diseases are age-related disorders with a high incidence and without disease-modifying treatment. Therefore, the evaluation of possible interventions that may affect disease progression is of paramount importance. Promising results from studies with GLP-1RAs in animal models have led to the use of these agents in human clinical trials.

Based on human trials in AD, the primary findings highlighted the positive impact of GLP-1RAs on cerebral glucose metabolism and a metabolic–neuroprotective effect. However, numerous problems limited the results obtained. Small study groups, early termination, and heterogeneous methodologies were significant limitations and could be the reason why clinical improvements were also not observed in the majority of studies. Future research needs to recruit larger cohorts, standardize the methodology, and introduce longer observation to confirm if GLP-1RAs present efficiency in AD therapy.

In clinical trials involving patients with PD, preliminary results indicate a short-term improvement in both motor and non-motor symptoms. There is also some evidence suggesting a potential disease-modifying effect; however, current data remain insufficient to confirm this hypothesis, particularly given the heterogeneity of dosing regimens and treatment durations. Future studies should be standardized, conducted over a long time, and designed to clarify whether the observed benefits arise from the optimization of neurotransmission via the GLP-1 pathway—reflecting a purely symptomatic effect—or whether they represent a true disease-modifying therapeutic action.

## Figures and Tables

**Table 1:** Inclusion and exclusion criteria selection of articles in review.

Inclusion Criteria	Exclusion Criteria
Articles in English language.	Research types of case studies, case series, reviews, editorials, conference abstracts, books, opinion articles, etc.
The research type of clinical trial is a randomized controlled trial.	Duplicate items.
Studies describing the use of GLP-1RA in the treatment of patients with neurodegenerative diseases, including Parkinson’s disease, atypical Parkinsonisms, including Multiple System Atrophy, Dementia with Lewy Bodies, Corticobasal Dementia, Progressive Supranuclear Palsy, Alzheimer’s disease, and Frontotemporal Dementia.	Studies describing the use of GLP-1RA in animals or cell experiments.

**Table 2:** Description of the study group and results in GLP-1RAs clinical trials in AD patients.

Number	Citation	Study Group, (*n*)	Age, Years	Intervention (Drug and Dose)	Clinical Trial Duration	Results
1	Gejl et al., 2016 [27]	Liraglutide (*n* = 18) Placebo (*n* = 20)	Placebo 66.6 ± 1.8 Liraglutide 63.1 ± 1.3	0.6 mg (1 week) ⟶ 1.2 mg (1 week) ⟶ 1.8 mg (thereafter) s.c. liraglutide	26 weeks	Liraglutide prevented the decline in CMRglc seen with placebo (significant decreases in precuneus, parietal, temporal, occipital, and cerebellum). In the liraglutide group, CMRglc rose slightly but not significantly. No group differences in amyloid deposition or cognition.
2	Gejl et al., 2017 [41]	Liraglutide (*n* = 18) Placebo (*n* = 20)	Placebo 66.6 ± 1.8 Liraglutide 63.1 ± 1.3	0.6 mg (1 week) ⟶ 1.2 mg (1 week) ⟶ 1.8 mg (thereafter) s.c. liraglutide	26 weeks	At baseline, CMRglc correlated positively with cognition and inversely with AD duration. Liraglutide increased Tmax from 0.72 to 1.1 μmol/g/min, reaching healthy control levels (*p* < 0.0001). No change in placebo (*p* = 0.24).
3	Watson et al., 2019 [42]	Liraglutide (*n* = 22) Placebo (*n* = 21)	Placebo 60.3 ± 5.4, 14 females Liraglutide 61.4 ± 6.1, 14 females	0.6 mg (1 week) ⟶ 1.2 mg (1 week) ⟶ 1.8 mg (thereafter) s.c. liraglutide	12 weeks	Higher fasting plasma glucose was linked to reduced hippocampal–frontal connectivity. After 6 months, the active group showed improved default mode network connectivity vs. placebo, with no cognitive differences. Glucose tolerance declined slightly more with liraglutide (*p* = 0.06).
4	Mullins et al., 2019 [43]	Exenatide (*n* = 14) Placebo (*n* = 13)	Placebo 74.0 ± 6.4 Exenatide 71.7 ± 6.9	Participants received s.c. exenatide 5 mcg twice daily (or placebo) for 1 week, then 10 mcg twice daily for 78 weeks.	18 months	Exenatide was safe and well-tolerated in MCI/early AD. Neuropsychological outcomes were largely similar, except for improved digit-span forward at 6 months. After 18 months, no group differences in GM atrophy or fluid biomarkers, except for a decrease in EV Aβ42 with exenatide (*p* = 0.045).
5	Dei Cas et al., 2024 [44]	Exenatide (*n* = 17) Placebo (*n* = 15)	Placebo 72 ± 6 Exenatide 74 ± 4	Long-acting exenatide 2 mg SC once a week.	32 weeks	No significant effect of exenatide on ADAS-Cog11 (*p* = 0.17). A gender interaction was observed (*p* = 0.04): women on exenatide showed cognitive decline (*p* = 0.018). Exenatide reduced fasting glucose (*p* = 0.02) and body weight (*p* = 0.03).

**Table 3:** Ongoing GLP-1RAs clinical trials in AD.

Number	Condition	Title	ClinicalTrials.gov ID	Phase	Status	Intervention (Drug and Dose)	Clinical Trial Duration
1	Early Alzheimer’s disease	A Research Study Investigating Semaglutide in People With Early Alzheimer’s Disease (EVOKE)	NCT04777396	Phase 3	Active, not recruiting	Semaglutide PO once a day, dose gradually increased to 14 mg.	173 weeks
2	Early Alzheimer’s disease	A Research Study Investigating Semaglutide in People With Early Alzheimer’s Disease (EVOKE Plus)	CT04777409	Phase 3	Active, not recruiting	Semaglutide PO once a day, dose gradually increased to 14 mg.	173 weeks
4	Alzheimer’s disease	A Research Study Looking at the Effect of Semaglutide on the Immune System and Other Biological Processes in People With Alzheimer’s Disease	NCT05891496	Phase 3	Active, not recruiting	Semagllutide SC 0.25 mg–1 mg once a week.	12 weeks
5	Alzheimer’s disease	Evaluating Liraglutide in Alzheimer’s Disease	NCT01843075	Phase 2	Unknown status	Liraglutide 1.8 mg once a day.	12 months

## Data Availability

The data supporting the findings of this study are available from the corresponding author upon reasonable request.

## References

[1] Holst J.J. (2007). The physiology of glucagon-like peptide 1. Physiol. Rev..

[2] Andersen A., Lund A., Knop F.K., Vilsbøll T. (2018). Glucagon-like peptide 1 in health and disease. Nat. Rev. Endocrinol..

[3] Reed J., Bain S., Kanamarlapudi V. (2020). Recent advances in understanding the role of glucagon-like peptide 1. F1000Research.

[4] Nadkarni P., Chepurny O.G., Holz G.G. (2014). Regulation of glucose homeostasis by GLP-1. Prog. Mol. Biol. Transl. Sci..

[5] Darsalia V., Larsson M., Nathanson D., Klein T., Nyström T., Patrone C. (2015). Glucagon-like receptor 1 agonists and DPP-4 inhibitors: Potential therapies for the treatment of stroke. J. Cereb. Blood Flow Metab. Off. J. Int. Soc. Cereb. Blood Flow Metab..

[6] Shannon R.P. (2013). DPP-4 inhibition and neuroprotection: Do mechanisms matter?. Diabetes.

[7] Ayoub B.M., Mowaka S., Safar M.M., Ashoush N., Arafa M.G., Michel H.E., Tadros M.M., Elmazar M.M., Mousa S.A. (2018). Repositioning of Omarigliptin as a once-weekly intranasal Anti-parkinsonian Agent. Sci. Rep..

[8] Daniels D., Mietlicki-Baase E.G. (2019). Glucagon-Like Peptide 1 in the Brain: Where Is It Coming From, Where Is It Going?. Diabetes.

[9] Campbell J.E., Drucker D.J. (2013). Pharmacology, physiology, and mechanisms of incretin hormone action. Cell Metab..

[10] Gupta T., Kaur M., Shekhawat D., Aggarwal R., Nanda N., Sahni D. (2023). Investigating the Glucagon-like Peptide-1 and Its Receptor in Human Brain: Distribution of Expression, Functional Implications, Age-related Changes and Species Specific Characteristics. Basic Clin. Neurosci..

[11] Hansen H.H., Fabricius K., Barkholt P., Niehoff M.L., Morley J.E., Jelsing J., Pyke C., Knudsen L.B., Farr S.A., Vrang N. (2015). The GLP-1 Receptor Agonist Liraglutide Improves Memory Function and Increases Hippocampal CA1 Neuronal Numbers in a Senescence-Accelerated Mouse Model of Alzheimer’s Disease. J. Alzheimer’s Dis. JAD.

[12] Li Y., Duffy K.B., Ottinger M.A., Ray B., Bailey J.A., Holloway H.W., Tweedie D., Perry T., Mattson M.P., Kapogiannis D. (2010). GLP-1 receptor stimulation reduces amyloid-beta peptide accumulation and cytotoxicity in cellular and animal models of Alzheimer’s disease. J. Alzheimer’s Dis. JAD.

[13] Li Y., Perry T., Kindy M.S., Harvey B.K., Tweedie D., Holloway H.W., Powers K., Shen H., Egan J.M., Sambamurti K. (2009). GLP-1 receptor stimulation preserves primary cortical and dopaminergic neurons in cellular and rodent models of stroke and Parkinsonism. Proc. Natl. Acad. Sci. USA.

[14] Chopade P., Chopade N., Zhao Z., Mitragotri S., Liao R., Chandran Suja V. (2022). Alzheimer’s and Parkinson’s disease therapies in the clinic. Bioeng. Transl. Med..

[15] Khan S., Barve K.H., Kumar M.S. (2020). Recent Advancements in Pathogenesis, Diagnostics and Treatment of Alzheimer’s Disease. Curr. Neuropharmacol..

[16] Malkiewicz J.J., Siuda J. (2024). Comparison of autonomic dysfunction in patients with Parkinson’s Disease, progressive supranuclear palsy, and multiple system atrophy. Neurol. I Neurochir. Pol..

[17] Balestrino R., Schapira A.H.V. (2020). Parkinson disease. Eur. J. Neurol..

[18] Cullinane P.W., de Pablo Fernandez E., König A., Outeiro T.F., Jaunmuktane Z., Warner T.T. (2023). Type 2 Diabetes and Parkinson’s Disease: A Focused Review of Current Concepts. Mov. Disord. Off. J. Mov. Disord. Soc..

[19] Cheong J.L.Y., de Pablo-Fernandez E., Foltynie T., Noyce A.J. (2020). The Association Between Type 2 Diabetes Mellitus and Parkinson’s Disease. J. Park. Dis..

[20] Chmiela T., Jarosz-Chobot P., Gorzkowska A. (2024). Glucose Metabolism Disorders and Parkinson’s Disease: Coincidence or Indicator of Dysautonomia?. Healthcare.

[21] Chmiela T., Węgrzynek J., Kasprzyk A., Waksmundzki D., Wilczek D., Gorzkowska A. (2022). If Not Insulin Resistance so What?—Comparison of Fasting Glycemia in Idiopathic Parkinson’s Disease and Atypical Parkinsonism. Diabetes Metab. Syndr. Obes. Targets Ther..

[22] Marques A., Dutheil F., Durand E., Rieu I., Mulliez A., Fantini M.L., Boirie Y., Durif F. (2018). Glucose dysregulation in Parkinson’s disease: Too much glucose or not enough insulin?. Park. Relat. Disord..

[23] Athauda D., Foltynie T. (2016). The glucagon-like peptide 1 (GLP) receptor as a therapeutic target in Parkinson’s disease: Mechanisms of action. Drug Discov. Today.

[24] Athauda D., Maclagan K., Skene S.S., Bajwa-Joseph M., Letchford D., Chowdhury K., Hibbert S., Budnik N., Zampedri L., Dickson J. (2017). Exenatide once weekly versus placebo in Parkinson’s disease: A randomised, double-blind, placebo-controlled trial. Lancet.

[25] Carranza-Naval M.J., Del Marco A., Hierro-Bujalance C., Alves-Martinez P., Infante-Garcia C., Vargas-Soria M., Herrera M., Barba-Cordoba B., Atienza-Navarro I., Lubian-Lopez S. (2021). Liraglutide Reduces Vascular Damage, Neuronal Loss, and Cognitive Impairment in a Mixed Murine Model of Alzheimer’s Disease and Type 2 Diabetes. Front. Aging Neurosci..

[26] Xie Y., Zheng J., Li S., Li H., Zhou Y., Zheng W., Zhang M., Liu L., Chen Z. (2021). GLP-1 improves the neuronal supportive ability of astrocytes in Alzheimer’s disease by regulating mitochondrial dysfunction via the cAMP/PKA pathway. Biochem. Pharmacol..

[27] Gejl M., Gjedde A., Egefjord L., Møller A., Hansen S.B., Vang K., Rodell A., Brændgaard H., Gottrup H., Schacht A. (2016). In Alzheimer’s Disease, 6-Month Treatment with GLP-1 Analog Prevents Decline of Brain Glucose Metabolism: Randomized, Placebo-Controlled, Double-Blind Clinical Trial. Front. Aging Neurosci..

[28] Zhang Y., Tang C., He Y., Zhang Y., Li Q., Zhang T., Zhao B., Tong A., Zhong Q., Zhong Z. (2024). Semaglutide ameliorates Alzheimer’s disease and restores oxytocin in APP/PS1 mice and human brain organoid models. Biomed. Pharmacother..

[29] Ma C., Hong F., Yang S. (2022). Amyloidosis in Alzheimer’s Disease: Pathogeny, Etiology, and Related Therapeutic Directions. Molecules.

[30] Yoon G., Kim Y.K., Song J. (2020). Glucagon-like peptide-1 suppresses neuroinflammation and improves neural structure. Pharmacol. Res..

[31] Yang S., Zhao X., Zhang Y., Tang Q., Li Y., Du Y., Yu P. (2024). Tirzepatide shows neuroprotective effects via regulating brain glucose metabolism in APP/PS1 mice. Peptides.

[32] Aksoy D., Solmaz V., Çavuşoğlu T., Meral A., Ateş U., Erbaş O. (2017). Neuroprotective Effects of Exenatide in a Rotenone-Induced Rat Model of Parkinson’s Disease. Am. J. Med. Sci..

[33] Bu L.L., Liu Y.Q., Shen Y., Fan Y., Yu W.B., Jiang D.L., Tang Y.L., Yang Y.J., Wu P., Zuo C.T. (2021). Neuroprotection of Exendin-4 by Enhanced Autophagy in a Parkinsonian Rat Model of α-Synucleinopathy. Neurotherapeutics.

[34] Zhu H., Zhang Y., Shi Z., Lu D., Li T., Ding Y., Ruan Y., Xu A. (2016). The Neuroprotection of Liraglutide Against Ischaemia-induced Apoptosis through the Activation of the PI3K/AKT and MAPK Pathways. Sci. Rep..

[35] Briyal S., Shah S., Gulati A. (2014). Neuroprotective and anti-apoptotic effects of liraglutide in the rat brain following focal cerebral ischemia. Neuroscience.

[36] Wang V., Tseng K.Y., Kuo T.T., Huang E.Y., Lan K.L., Chen Z.R., Ma K.H., Greig N.H., Jung J., Choi H.I. (2024). Attenuating mitochondrial dysfunction and morphological disruption with PT320 delays dopamine degeneration in MitoPark mice. J. Biomed. Sci..

[37] Tian R., Liu K., Lai H., Liao C., Li J., Tu H. (2025). GLP-1/GIP dual agonist tirzepatide alleviates mice model of Parkinson’s disease by promoting mitochondrial homeostasis. Int. Immunopharmacol..

[38] Song D., Zou X., Ma D., Zhao Y., Liu T., Shen B., Cheng O. (2025). The GLP1R Agonist Semaglutide Inhibits Reactive Astrocytes and Enhances the Efficacy of Neural Stem Cell Transplantation Therapy in Parkinson’s Disease Mice. Adv. Sci..

[39] Cai H.Y., Hölscher C., Yue X.H., Zhang S.X., Wang X.H., Qiao F., Yang W., Qi J.S. (2014). Lixisenatide rescues spatial memory and synaptic plasticity from amyloid β protein-induced impairments in rats. Neuroscience.

[40] McClean P.L., Hölscher C. (2014). Liraglutide can reverse memory impairment, synaptic loss and reduce plaque load in aged APP/PS1 mice, a model of Alzheimer’s disease. Neuropharmacology.

[41] Gejl M., Brock B., Egefjord L., Vang K., Rungby J., Gjedde A. (2017). Blood-Brain Glucose Transfer in Alzheimer’s disease: Effect of GLP-1 Analog Treatment. Sci. Rep..

[42] Watson K.T., Wroolie T.E., Tong G., Foland-Ross L.C., Frangou S., Singh M., McIntyre R.S., Roat-Shumway S., Myoraku A., Reiss A.L. (2019). Neural correlates of liraglutide effects in persons at risk for Alzheimer’s disease. Behav. Brain Res..

[43] Mullins R.J., Mustapic M., Chia C.W., Carlson O., Gulyani S., Tran J., Li Y., Mattson M.P., Resnick S., Egan J.M. (2019). A Pilot Study of Exenatide Actions in Alzheimer’s Disease. Curr. Alzheimer Res..

[44] Dei Cas A., Micheli M.M., Aldigeri R., Gardini S., Ferrari-Pellegrini F., Perini M., Messa G., Antonini M., Spigoni V., Cinquegrani G. (2024). Long-acting exenatide does not prevent cognitive decline in mild cognitive impairment: A proof-of-concept clinical trial. J. Endocrinol. Investig..

[45] Weihe E., Depboylu C., Schütz B., Schäfer M.K., Eiden L.E. (2006). Three types of tyrosine hydroxylase-positive CNS neurons distinguished by dopa decarboxylase and VMAT2 co-expression. Cell. Mol. Neurobiol..

[46] Aviles-Olmos I., Dickson J., Kefalopoulou Z., Djamshidian A., Ell P., Soderlund T., Whitton P., Wyse R., Isaacs T., Lees A. (2013). Exenatide and the treatment of patients with Parkinson’s disease. J. Clin. Investig..

[47] Athauda D., Maclagan K., Budnik N., Zampedri L., Hibbert S., Aviles-Olmos I., Chowdhury K., Skene S.S., Limousin P., Foltynie T. (2019). Post hoc analysis of the Exenatide-PD trial-Factors that predict response. Eur. J. Neurosci..

[48] Athauda D., Maclagan K., Budnik N., Zampedri L., Hibbert S., Skene S.S., Chowdhury K., Aviles-Olmos I., Limousin P., Foltynie T. (2018). What Effects Might Exenatide have on Non-Motor Symptoms in Parkinson’s Disease: A Post Hoc Analysis. J. Park. Dis..

[49] Athauda D., Gulyani S., Karnati H.K., Li Y., Tweedie D., Mustapic M., Chawla S., Chowdhury K., Skene S.S., Greig N.H. (2019). Utility of Neuronal-Derived Exosomes to Examine Molecular Mechanisms That Affect Motor Function in Patients with Parkinson Disease: A Secondary Analysis of the Exenatide-PD Trial. JAMA Neurol..

[50] McGarry A., Rosanbalm S., Leinonen M., Olanow C.W., To D., Bell A., Lee D., Chang J., Dubow J., Dhall R. (2024). Safety, tolerability, and efficacy of NLY01 in early untreated Parkinson’s disease: A randomised, double-blind, placebo-controlled trial. Lancet Neurol..

[51] Meissner W.G., Remy P., Giordana C., Maltête D., Derkinderen P., Houéto J.L., Anheim M., Benatru I., Boraud T., Brefel-Courbon C. (2024). LIXIPARK Study Group Trial of Lixisenatide in Early Parkinson’s Disease. N. Engl. J. Med..

[52] Lo R.Y. (2021). Epidemiology of atypical parkinsonian syndromes. Tzu Chi Med. J..

[53] Liu M., Wang Z., Shang H. (2024). Multiple system atrophy: An update and emerging directions of biomarkers and clinical trials. J. Neurol..

[54] Koekkoek P.S., Kappelle L.J., van den Berg E., Rutten G.E., Biessels G.J. (2015). Cognitive function in patients with diabetes mellitus: Guidance for daily care. Lancet Neurol..

[55] Wang W., Wang Q., Qi X., Gurney M., Perry G., Volkow N.D., Davis P.B., Kaelber D.C., Xu R. (2024). Associations of semaglutide with first-time diagnosis of Alzheimer’s disease in patients with type 2 diabetes: Target trial emulation using nationwide real-world data in the US. Alzheimer’s Dement. J. Alzheimer’s Assoc..

[56] Song S., Luo Z., Li C., Huang X., Shiroma E.J., Simonsick E.M., Chen H. (2021). Changes in Body Composition Before and After Parkinson’s Disease Diagnosis. Mov. Disord. Off. J. Mov. Disord. Soc..

[57] Camacho M., Greenland J.C., Williams-Gray C.H. (2021). The Gastrointestinal Dysfunction Scale for Parkinson’s Disease. Mov. Disord. Off. J. Mov. Disord. Soc..

[58] Węgrzynek-Gallina J., Chmiela T., Kasprzyk A., Borończyk M., Siuda J. (2025). Metabolic effects of deep brain stimulation in Parkinson’s disease—A systematic review and meta-analysis. Neurol. I Neurochir. Pol..

[59] Zhang C., Xu J., Wu B., Ling Y., Guo Q., Wang S., Liu L., Jiang N., Chen L., Liu J. (2022). Subthalamic Nucleus Deep Brain Stimulation Treats Parkinson’s Disease Patients with Cardiovascular Disease Comorbidity: A Retrospective Study of a Single Center Experience. Brain Sci..

